# Functions of TIP60/NuA4 Complex Subunits in Cell Differentiation

**DOI:** 10.3390/cells14211720

**Published:** 2025-11-01

**Authors:** Fatemeh Hashemi, Aida Nourozi, Mojtaba Shaban Loushab, Karl Riabowol

**Affiliations:** 1 Arnie Charbonneau Cancer Institute, Cumming School of Medicine, University of Calgary, Calgary, AB T2N 4N1, Canada; fatemeh.hashemi1@ucalgary.ca (F.H.); shaban.mojtaba2014@gmail.com (M.S.L.); 2 Departments of Biochemistry & Molecular Biology, Cumming School of Medicine, University of Calgary, Calgary, AB T2N 4N1, Canada; 3 Division of Genome Sciences and Cancer, The Shine Dalgarno Centre for RNA Innovation, The John Curtin School of Medical Research, The Australian National University, Canberra 2601, Australia; aida.norouzi@anu.edu.au; 4 Robson DNA Sciences Centre, Cumming School of Medicine, University of Calgary, Calgary, AB T2N 4N1, Canada; 5 Department of Oncology, Cumming School of Medicine, University of Calgary, Calgary, AB T2N 4N1, Canada

**Keywords:** TIP60/NuA4 complex, histone acetyltransferase, ING3, stem cells, differentiation

## Abstract

**Highlights:**

**What are the main findings?**

- TIP60/NuA4 functions as an integrated complex—catalytic (TIP60/KAT5), scaffold/ATPase (EP400/EPC1/TRRAP), and reader subunits (ING3, YEATS4, MBTD1, BAF53A/MRG15) cooperate to remodel chromatin during lineage-specific differentiation.
- Individual subunits have distinct yet convergent roles across neurogenesis, myogenesis, adipogenesis, hematopoiesis, and germ-cell development; viewing the whole complex explains overlapping differentiation phenotypes.

**What are the implications of the main findings?**

- A whole-complex perspective clarifies why disruption of different subunits yields similar outcomes and helps separate complex-dependent from subunit-specific effects.
- TIP60/NuA4 emerges as a central epigenetic hub with translational relevance for disorders of development, regeneration, and disease.

**Abstract:**

The TIP60/NuA4 complex is a large, multifunctional histone acetyltransferase assembly of ~1.7 megadaltons, composed of 17–20 subunits, which plays a central role in epigenetic regulation. Through recognition of H3K4me3 by the ING3 reader, TIP60/NuA4 is recruited to sites of active transcription, where it remodels chromatin to regulate gene expression. Its activities include histone acetylation, histone variant exchange, transcriptional co-activation, and regulation of the cell cycle and apoptosis. In this review, we examine how altered subunit levels or mutations impact the chromatin structure and transcriptional activity, and how these changes influence differentiation across diverse cell types. We emphasize the molecular mechanisms by which TIP60/NuA4 shapes lineage specification, including histone H2A and H4 acetylation by the KAT5 catalytic subunit, H2A.Z incorporation by EP400, and interactions with transcription factors such as MyoD, PPARγ, and Myc. By integrating mechanistic and functional insights, we highlight how TIP60/NuA4 acts as a central epigenetic hub in differentiation and contributes to proper developmental transitions.

## 1. Introduction

All cells in an organism originate from pre-existing cells, beginning with the fertilized egg and progressing to the blastocyst stage. Stem cells, possessing totipotency, have the capacity to differentiate into any cell type, including placental cells. Their key characteristics, proliferation and differentiation, enable them to multiply and specialize. The programming of stem cells relies heavily on epigenetic regulation to guide lineage-specific differentiation, which is especially critical for generating induced pluripotent stem cells (iPSCs) for applications such as cell replacement therapy [1]. Epigenetic regulatory proteins function at distinct stages of differentiation, guiding stem cells toward lineage commitment. These finely tuned processes are crucial not only for development but also for advancing therapeutic strategies [1].

Epigenetics refers to heritable changes in gene expression that occur without altering the DNA sequence. Key epigenetic mechanisms include DNA cytosine methylation, ATP-dependent nucleosome remodeling, histone variant substitution, numerous histone modifications, and regulation of gene expression by small non-coding RNAs [2]. As stem cells begin to differentiate, epigenetic mechanisms undergo a significant shift—from initially suppressing genes associated with differentiation to actively promoting the expression of genes essential for specific cellular fates [3]. Dysregulation of this process is frequently implicated in diseases such as cancer.

Histone modifications involve numerous post-translational changes, including acetylation, methylation, phosphorylation, ubiquitination, sumoylation, formylation, butyrylation, and others [4]. Lysine residues within the core nucleosomal histones are the targets of many modifications, with acetylation playing a major role in regulating nucleosome structure. Histone acetyltransferases (HATs, also called lysine acetyltransferases or KATs), including members of the p300/CBP, MYST (such as MOZ, Sas2, TIP60), and GNAT families, catalyze the acetylation of both histone and non-histone proteins [5]. In stem cells, HATs play dual roles by maintaining pluripotency and promoting differentiation. By selectively activating or repressing gene expression, HAT complexes orchestrate the balance between self-renewal and differentiation [6].

The MYST family of HATs, including MOF and MYST2/HBO1, exerts a significant influence on gene expression by acetylating histones to reshape chromatin structure and direct transcriptional patterns [6]. Among these, the TIP60/NuA4 complex, a key member of the MYST family, is a major regulator of the epigenetic landscape controlling stem cell behavior.

**Figure 1:**
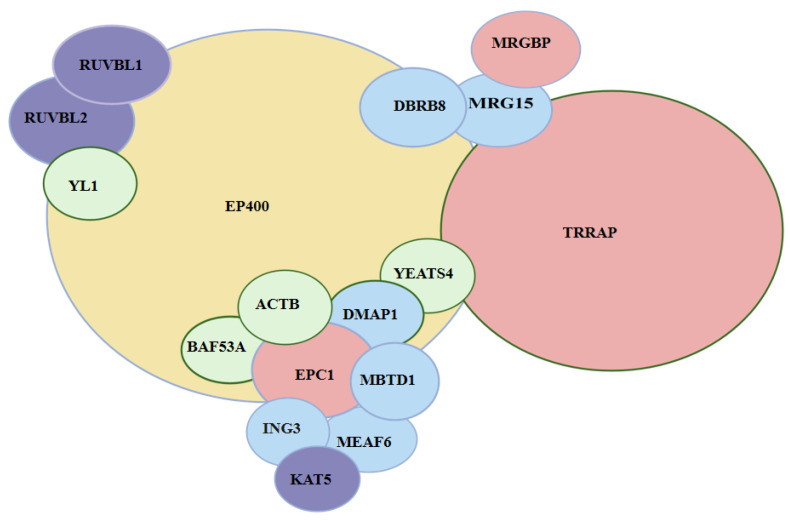
TIP60 Complex Members. Schematic representation of the TIP60/NuA4 chromatin remodeling and histone acetyltransferase complex, with subunits grouped by functional role. The central yellow circle represents p400 (EP400), the primary scaffold of the complex with ATPase activity. Light red components depict scaffold subunits (EPC1, MRGBP, and TRRAP) that stabilize and organize the assembly [7,9]. Purple components indicate enzymatic/catalytic members (KAT5, RUVBL1, RUVBL2) responsible for histone acetyltransferase and ATPase activities. Light blue components represent accessory/regulatory subunits (DMAP1, ING3, MEAF6, MRG15, MBTD1, and BRD8) that modulate activity, target specificity, and complex stability. Light green members represent the structural core of the complex (ACTL6A (BAF53A), VPS72 (YL1), and YEATS4 (GAS41)) [7,9].

The human TIP60 complex is a large assembly (>1.7 megadaltons) composed of 17–20 subunits, including ACTB, ACTL6A, BRD8, DMAP1, EP400, EPC1, ING3, KAT5, MBTD1, MEAF6, MRG15, MRGBP, RUVBL1, RUVBL2, TRRAP, YEATS4 and YL1 (Figure 1) [7]. The primary subunits, their aliases, and functional categories are summarized in Table 1. This complex regulates transcription, proliferation, and homologous recombination (HR)-mediated DNA repair. KAT5 acetylates H2A, H2A.Z, and H4, and the complex facilitates H2A.Z incorporation via EP400’s ATP-dependent remodeling activity. Given these diverse roles, the TIP60/NuA4 complex is vital for maintaining stem cell renewal and differentiation potential [8].

**Figure 2:**
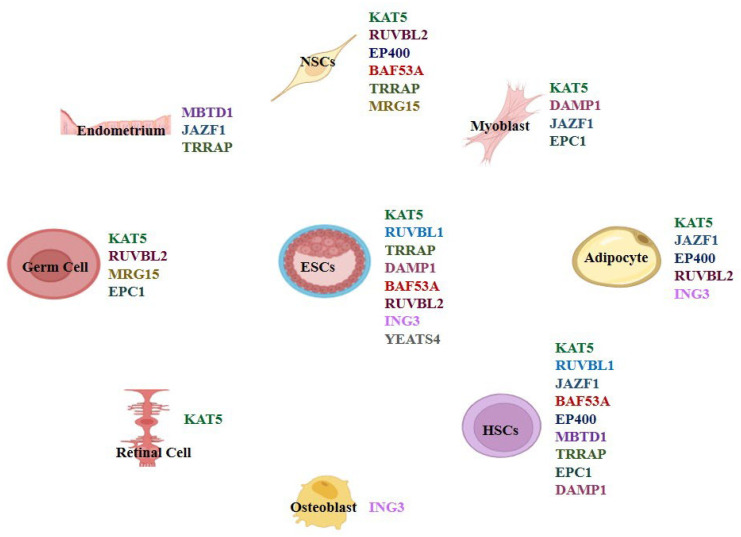
Components of the TIP60/NuA4 Complex Linked to Lineage-Specific Cell Differentiation. References cited present evidence for a functional role of the indicated TIP60/NuA4 complex member in the form of differentiation noted by the label. For example, ING3 knockdown was reported to inhibit osteoblastogenesis and promote adipogenesis [10] and is implicated in reprogramming of epigenetic memory in oocytes of different species [11] and of suppressing endogenous retroviral elements in early development [12].

**Figure 3:**
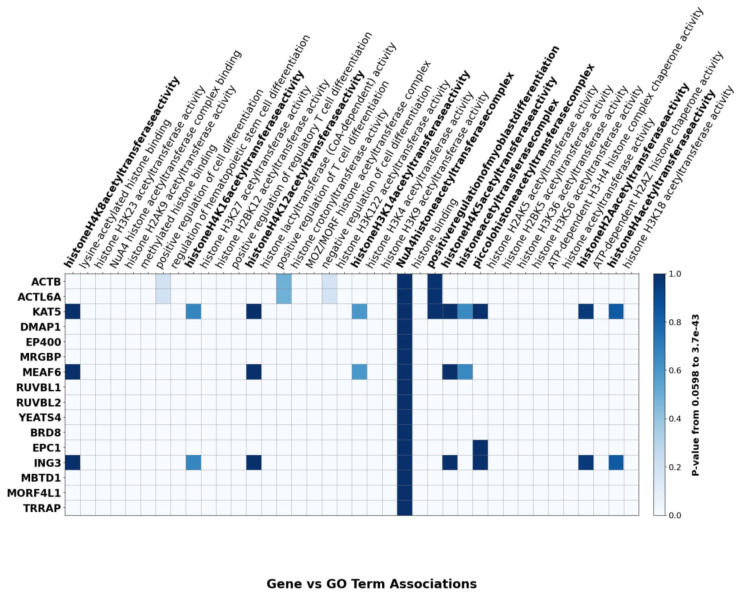
Heatmap of TIP60/NuA4 Complex Members vs. GO Term Associations Based on Enrichment Significance. Each cell indicates the association strength between a TIP60/NuA4 subunit (*y*-axis) and a GO term (*x*-axis). Color intensity reflects enrichment significance (darker blue = lower *p*-value). Bolded GO terms denote key biological functions such as histone acetyltransferase activity and chromatin regulation. Enrichment was computed against the human proteome background with FDR correction (Benjamini–Hochberg); *p*-values shown are adjusted (range: 0.0598 to 3.71 × 10^−43^).

The differential roles of TIP60/NuA4 complex subunits in lineage-specific differentiation are summarized in Figure 2, which identifies the individual components that have been reported to contribute to the developmental pathways noted. A heatmap of TIP60 subunit–GO term associations further emphasizes biological processes involving TIP60 and its subunits, including histone acetyltransferase activity and chromatin regulation (Figure 3).

While several recent reviews have discussed general functions of TIP60/NuA4 in DNA repair, transcription, and development, our review takes a distinct approach by examining the lineage-specific roles of individual subunits in cell differentiation. By integrating evidence across multiple cell types, we provide a subunit-level perspective that highlights both shared and unique contributions to chromatin remodeling. This framework allows us to not only summarize existing knowledge but also identify conceptual gaps, controversies, and translational opportunities that extend beyond prior overviews.

## 2. Functions of TIP60/NuA4 Subunits in Differentiation

### 2.1. TIP60/KAT5 and Differentiation

KAT5, the catalytic subunit of the TIP60/NuA4 complex, is a 60 kD lysine acetyltransferase subunit that is evolutionarily conserved from yeast to humans. TIP60/KAT5 interacts with numerous transcription factors and co-regulators, playing critical roles in apoptosis, DNA damage response, cell cycle control, and transcriptional regulation [13]. It functions as a co-activator for transcription factors such as HIV Tat and MyoD, facilitating histone acetylation at target promoters. It also interacts with CREB, STAT3, ZEB, and p73, further influencing gene expression [13,14].

TIP60/KAT5 is essential in multiple differentiation processes, including neurodevelopment, hematopoiesis, germ cell maturation, adipogenesis, and myogenesis (Figure 2). In the sections that follow, we examine TIP60/KAT5’s roles in the context of lineage-specific differentiation.

TIP60/KAT5 and neuronal differentiation. Studies using neural stem cell (NSC)-specific TIP60/KAT5 knockout mice have shown that TIP60/KAT5 is critical for neurodevelopment. Its absence leads to disrupted NSC proliferation, microcephaly, impaired neuronal migration, and increased GFAP-positive glial cells. TIP60/KAT5 is required for neurogenesis, NSC maintenance, and the neurogenesis-to-gliogenesis transition [13].

TIP60/KAT5 is recruited to chromatin through its interaction with neuronal lineage–determining transcription factors, which enables histone H2A.Z acetylation and promotes neuronal fate [15]. Bivalent chromatin domains play a key role in regulating development in pluripotent stem cells, marking important gene promoters and enhancers ready for activation. Recent studies suggest that TIP60/KAT5-mediated H2A.Z acetylation might influence the transition from bivalency to monovalent domains during differentiation, particularly in the activation of bivalent genes and the acquisition of H3K4me3 marks [15]. Increasing evidence suggests that H3K4me3 primarily prevents active repression rather than directly activating genes. Its absence leads to increased H3K27me3 and DNA methylation in these regions, disrupting differentiation, as genes involved in neuronal differentiation cannot be activated [15,16]. Mutations in the TIP60/KAT5 gene are associated with a neurodevelopmental syndrome characterized by progressive cerebellar atrophy, developmental delays, and intellectual disabilities [17,18].

Eichele et al. [19] reported parallels between TIP60/KAT5 deficiency and the CK-p25 neurodegeneration model, noting hypoacetylation of H4K12 and dysregulation of transcription in TIP60/KAT5-deficient mice. Genes linked to immunity and cell cycle were upregulated, while those associated with learning and plasticity were downregulated. This imbalance leads to caspase-3 activation, β-actin fragmentation in CA1 neurites, and neuronal loss [19]. Thus, TIP60/KAT5 emerges as a key guardian of neuronal health by mitigating early imbalances in TIP60/KAT5 HAT/histone deacetylase (HDAC) activity observed in various neurodegenerative diseases. When TIP60/HDAC homeostasis is disrupted, synaptic anomalies and cognitive decline emerge in the preclinical stages of these diseases. Elevating cellular levels of specific neural HATs, notably TIP60/KAT5, serves as a protective measure, restoring acetylation equilibrium and preserving the essential gene expression profiles vital for neuroplasticity and neural well-being [14,20].

TIP60/KAT5 and hematopoietic stem cells (HSCs). In hematopoiesis, TIP60/KAT5 plays a regulatory role in both normal development and malignancy. Chromosomal translocations in some acute myeloid leukemia (AML) cases produce a fusion gene known as ZMYND11-MBTD1 (ZM), which recruits TIP60/KAT5 to stemness-related gene regions, including MYC, HoxA, Meis1, and Sox4. This recruitment promotes continuous HSC self-renewal and contributes to leukemogenesis [21]. Using conditional knockout models, Daniel et al. demonstrated that TIP60/KAT5 is essential for regulating MYC target gene expression during both embryonic and adult stages of HSC maintenance. TIP60/KAT5 colocalizes with c-Myc, and its deletion suppresses genes responsible for cell cycle control and DNA repair; two fundamental processes for sustaining HSC function [22].

TIP60/KAT5 and germ cell differentiation. In female *drosophila* germ cells, TIP60/KAT5 functions in conjunction with Nipped-A to regulate the differentiation of germline stem cells (GSCs). GSCs maintain the ability to self-renew or differentiate, and genes such as bag of marbles (bam) and benign gonial cell neoplasm (bgcn) are key to this process. Nipped-A, through its association with the TIP60/NuA4 complex, promotes cell cycle progression and the expression of bgcn, facilitating the transition of GSC daughter cells toward differentiation. Loss of Nipped-A results in an accumulation of undifferentiated GSC daughters, a defect that can be rescued by bgcn overexpression. This highlights TIP60/KAT5’s role in coordinating cell cycle regulation with differentiation cues in germ cells [23].

TIP60/KAT5 and spermiogenesis. During spermiogenesis, the final phase of sperm development, TIP60/KAT5 plays an essential role. This process involves replacing histones with protamines to achieve chromatin condensation, and histone hyperacetylation is a critical preparatory step. Specifically, TIP60/KAT5 and its associated complex member Epc1 are necessary for this histone hyperacetylation. Disruption of either gene results in abnormal spermatid morphology and impaired chromatin remodeling, indicating important functional roles in genome compaction during male germ cell differentiation [24].

TIP60/KAT5 and embryonic stem cell (ESC) differentiation. ESCs are widely used to study pluripotency and differentiation. In the absence of TIP60-EP400 complex components, ESCs fail to differentiate properly, forming small, irregular embryoid bodies and lacking activation of lineage-specific markers [25]. In TIP60/KAT5-knockout mice, embryos exhibit abnormalities at the blastocyst stage, with impaired hatching and failure to progress post-implantation [26].

TIP60/KAT5 has both HAT-dependent and HAT-independent functions in embryonic stem cells. While its acetyltransferase activity is required for proper differentiation into mesoderm and endoderm, TIP60/KAT5 can also repress differentiation-associated genes by reducing chromatin accessibility at their promoters, independent of its enzymatic function. In this context, it maintains compact promoter regions through binding rather than acetylation. As a result, ESCs with TIP60/KAT5 mutations can still self-renew but show impaired differentiation. This dual role underscores the complex regulatory influence of the catalytic subunit on ESC fate [27].

TIP60/KAT5 and myoblasts. Myoblasts are precursor cells that commit to the skeletal muscle lineage and eventually fuse to form multinucleated myotubes. TIP60/KAT5 plays an essential role in this process by activating MyoD’s transcriptional activity, a key driver of myogenesis. In C2C12 myoblasts, knockdown of TIP60/KAT5 impaired the transition from proliferating myoblasts to differentiated myotubes, while ectopic TIP60/KAT5 expression enhanced MyoD-mediated gene activation. TIP60/KAT5 physically interacts with MyoD via its chromo- and Zn-finger domains, which are critical for effective transcriptional activation. This interaction facilitates MyoD binding to the myogenin promoter, an early marker of muscle differentiation, and promotes chromatin remodeling by increasing histone H3 and H4 acetylation at the promoter site [28].

In addition to its role in MyoD activation, TIP60/KAT5’s influence extends to the regulation of other transcription factors such as SOX4, which also play critical roles in myoblast differentiation. TIP60/KAT5 specifically acetylates SOX4 at lysine 95, enabling its binding to target gene promoters and the activation of muscle-specific genes. Furthermore, SOX4 initially associates with the co-repressor HDAC1, and during C2C12 differentiation, HDAC1 dissociates, allowing SOX4 to be acetylated by TIP60/KAT5. This acetylation is essential for the recruitment of SOX4 to the promoter of caldesmon 1, a gene involved in actin cytoskeleton organization and muscle contraction, ensuring its full transcriptional activation. This study identified a molecular switch between the co-activator TIP60/KAT5 and co-repressor HDAC1 that governs SOX4’s transcriptional activity for precisely regulating gene expression and muscle cell differentiation [29].

TIP60/KAT5 and adipogenesis. Peroxisome proliferator-activated receptor γ (PPARγ) is an important regulator in adipocytes, affecting adipogenesis, lipid storage, thermogenesis, and insulin sensitivity. In response to metabolic cues, PPARγ recruits different cofactors, forming dynamic transcriptional complexes for specific signal transduction [30]. In adipogenesis, TIP60/KAT5 enhances PPARγ activity by binding to the AF1 region of PPARγ2, which is critical for adipocyte-specific transcription. This coactivation requires TIP60/KAT5’s histone acetyltransferase activity, since catalytic mutants show reduced stimulation of PPARγ-driven transcription. Chromatin immunoprecipitation also revealed that TIP60/KAT5 is recruited with PPARγ to adipogenic promoters during differentiation, supporting its role as an epigenetic coactivator through the TIP60/NuA4 complex [31]. Moreover, the stability of TIP60/KAT5, carefully maintained by ubiquitin-specific protease 7 (USP7), becomes especially important during the early stages of adipocyte development, particularly when cells undergo mitotic clonal expansion. TIP60’s dual role thus spans both early and later phases of fat cell maturation [32].

TIP60/KAT5 and retinal differentiation. TIP60/KAT5 functions as a co-activator in the transcriptional regulation of rod photoreceptor differentiation in the retina. TIP60/KAT5 interacts with Nrl, a bZIP transcription factor, to enhance expression of rod-specific genes like rhodopsin and Ppp2r5c. This interaction is facilitated by the phosphorylation of Nrl at Ser50 by JNK1, which enables TIP60/KAT5 to acetylate histones at the promoters of these genes, thereby promoting their transcription [33].

### 2.2. ING3 and Differentiation

The INhibitor of Growth family of proteins (ING1–5) all function as stoichiometric targeting components of HAT and HDAC complexes by serving as epigenetic readers of the histone H3K4me3 mark through their plant homeodomain [34]. The ING1 and 2 proteins also act as receptors and transducers of stress-activated phosphoinositides, linking stress to transcriptional repression [35]. These repressive functions, through targeting the Sin3a HDAC complex, are likely to underlie their function as type II tumor suppressor genes due to their frequent inactivation in malignancies [36]. ING3 was initially discovered through an in silico computational approach [37]. Like other ING proteins [38], ING3 affects TP53-mediated transcription [39], exerting control over the cell cycle and apoptosis. ING3 exhibits elevated expression in cells undergoing rapid proliferation, suggesting a positive role in cellular growth and self-renewal [40]. ING3 is also required for development, where its loss causes severe growth restriction and embryonic death. The most pronounced effects are on ectoderm-derived tissues, resulting in a developmental defect in neural tube closure and the inability to form intact primary brain vesicles in mid-gestation homozygous knockout embryos [41].

ING3 and mesenchymal stem cell differentiation. A study conducted by Chesi et al. [10] showed that ING3 knockdown in mesenchymal stem cells reduces osteoblast differentiation, with decreased ALP activity and mineral deposition, while enhancing adipogenesis through increased C/EBPα expression as estimated by increased Oil Red O staining. Although this study did not analyze histone modifications or the involvement of other NuA4/TIP60 components, ING3 is known to recruit the TIP60/NuA4 complex to H3K4me3 through its PHD domain. Its role in osteogenesis may therefore involve epigenetic activation of osteogenic genes, but this has not been directly demonstrated, and whether the observed effects are NuA4-dependent remains to be determined [10].

ING3 and oocyte maturation. Beyond its role in mesenchymal stem cell and skeletal differentiation, ING3 is essential for oocyte maturation, a process defined by asymmetric cell division. It may exert this function through the mammalian target of rapamycin (mTOR) pathway, as ING3 knockdown decreases the expression of mTOR and its downstream targets, including cell division control protein 42 (Cdc42), Ras-related C3 botulinum toxin substrate 1 (Rac1), and Ras homolog family member A (RhoA). The absence of ING3 results in symmetric division, a feature commonly seen in aging mouse oocytes, highlighting its role in regulating genes needed for proper oocyte maturation [42].

### 2.3. MRG15 and Differentiation

MRG15 (MORF4-Related Gene on chromosome 15) is a conserved chromatin-associated protein that plays a key role in epigenetic regulation by associating with histone-modifying complexes such as TIP60. It contains an N-terminal chromodomain that binds to H3K36me2/3 (histone H3 lysine 36 di/trimethylation), linking it to transcriptionally active chromatin. Its C-terminal MRG domain mediates interactions with partners like hMOF, Rb, and MRGBP, facilitating gene regulation, DNA repair, and cell cycle control. MRG15 is essential for embryonic development and neural stem cell maintenance [43]. Depleting MRG15 in Drosophila causes embryonic lethality, hampers DNA repair, and disrupts cellular differentiation. In mice, Mrg15 null cells exhibit reduced proliferation, leading to early senescence [44].

MRG15 and neuronal differentiation. Chen et al. reported that MRG15 functions in Neural Precursor Cell (NPC) proliferation and differentiation during neurogenesis. Loss of MRG15 in embryonic brains leads to thinner neuroepithelia and increased apoptosis in vivo, while NPCs from Mrg15-deficient embryos show reduced proliferation and impaired neuronal differentiation in vitro, without increased apoptosis. These defects are likely due to MRG15’s role in chromatin remodeling through its association with TIP60 HAT and HDAC complexes, which regulate gene expression necessary for NPC fate decisions. Although MRG15 knockdown in ESCs shows minimal effects—possibly due to compensation by its homolog MRGX—its absence significantly affects tissue-specific precursor cells like NPCs. MRG15-deficient NPCs fail to properly attach and differentiate, suggesting cell-autonomous defects that may extend to other tissues [45].

MRG15 also helps maintain genomic stability by regulating the DNA damage response. Loss of MRG15 leads to activation of p53, upregulation of p21, increased 53BP1 DNA damage foci, and impaired DNA repair, even without external insults [46]. These effects may be mediated through disruption of the TIP60/NuA4 complex or the BRCA2-PALB2 pathway since both are involved in DNA repair. MRG15 may also suppress p21 expression through transcriptional repression mechanisms.

MRG15 and germ cell differentiation. Some researchers have shown that MRG-1, a protein containing a chromodomain and a homolog of mammalian MRG15 helps balance stem cell proliferation and differentiation in *C. elegans*. Proteasome-mediated protein degradation regulates MRG-1 levels, and its interaction with the E3 ubiquitin ligase RFP-1 is involved in this regulation. Reduced proteasome activity or loss of RFP-1 expression leads to elevated MRG-1 levels and stem cell over-proliferation while MRG-1 absence results in germ cell loss. This suggests that MRG-1 likely influences the expression of proteins necessary for proliferation and represses those necessary for differentiation, thereby affecting the balance between the two processes. Notably, MRG15 exhibits similar functions in murine neurospheres suggesting conservation of its role in regulating stem cell proliferation and differentiation across species [47].

MRG15 also functions during spermatogenesis since MRG15 loss in postnatal male germ cells results in spermatogenesis being blocked at the round spermatid stage. This arrest does not disrupt meiotic division or histone acetylation, but it exerts a significant influence on the epigenetic control of pre-mRNA splicing. Genes like transition nuclear protein 2 (Tnp2) are particularly affected by the absence of MRG15, leading to errors in splicing. These splicing errors cause an accumulation of multinucleated round spermatids and inflammation in the epididymides [48].

### 2.4. MBTD1 and Differentiation

Mbt Domain Containing 1 (MBTD1), also known as Hemp (hematopoietic expressed mammalian polycomb) is a nuclear protein with a Cys2-Cys2 zinc finger domain and four malignant brain tumor (MBT) repeats. Originally discovered in mouse fetal liver, MBTD1 is involved in regulating gene expression by binding methylated lysine residues, especially H4K20 [49]. In the context of DNA double-strand break (DSB) repair, MBTD1 competes with 53BP1, a DNA repair factor, for binding to the H4K20me mark at DSB sites and, in association with TIP60/KAT5, acetylates histone H4 and H2AK15. This destabilizes 53BP1 interactions and prevents its recruitment, ultimately directing the cellular response toward error-free HR repair of DSBs [50].

MBTD1 and endometrial stromal cell differentiation. MBTD1 affects differentiation during endometrial stromal cell decidualization. This differentiation process is needed for successful pregnancy since deficiencies in it can lead to early miscarriages. MBTD1 is upregulated during decidualization and functions in progesterone-driven human and mouse endometrial stromal cell differentiation by promoting DNA double-strand break repair, which is critical during decidualization when DNA damage levels can increase. This suggests that MBTD1 contributes to genomic stability during the differentiation process, potentially protecting against DNA damage [51].

MBTD1 and HSCs. MBTD1 also contributes to the development and maintenance of hematopoietic stem cells. During fetal development, it is highly expressed in early fetal liver HSCs, where it supports their proliferation, survival, and repopulating capacity. Loss of MBTD1 during this stage results in a significant reduction in HSC numbers, impaired colony formation, and decreased engraftment potential. These defects are associated with reduced cell proliferation, increased apoptosis, and dysregulation of key hematopoietic genes, including Sox4, Stat3, and KLF6 [52].

In adult HSCs, MBTD1 also helps preserve quiescence and long-term function. Conditional knockout studies in mice showed that MBTD1 promotes the expression of FOXO3a, a transcription factor that activates cell cycle inhibitors such as p57 and p21. Without MBTD1, HSCs lose quiescence, enter the cell cycle more frequently, and become less resilient to physiological stress. Proteomic analyses further indicate that MBTD1 interacts with several components of the NuA4/TIP60 chromatin remodeling complex, suggesting that its regulation of HSC fate may involve epigenetic mechanisms, although this has not been fully demonstrated [49].

### 2.5. EPC1 and Differentiation

EPC1 (enhancer of polycomb homolog 1) is a conserved chromatin regulator and a member of the polycomb group (PcG) protein family involved in epigenetic gene silencing and maintenance of cellular identity. As a non-catalytic but essential component of the NuA4/TIP60 histone acetyltransferase complex, EPC1 promotes transcription, DNA repair, and cell cycle regulation by coordinating histone acetylation. Its yeast homolog, Epl1, plays a similar role in chromatin remodeling. EPC1 also modulates gene expression as a transcriptional co-regulator of E2F1 and interacts with complexes like PRC2. While EPC1 is implicated in developmental processes such as hematopoiesis and muscle differentiation, it is also associated with cancer progression, suppressing apoptosis and contributing to drug resistance through interactions with TIP60 and EZH2 [53,54].

Studies in Drosophila development have illuminated the involvement of EPC1 in processes related to differentiation and the determination of stem cell fate. For example, activation of the JNK (Jun amino-terminal kinase) signaling pathway in imaginal disc cells during regeneration leads to the downregulation of EPC1, facilitating wound healing and contributing to the formation of major adult fly structures. Furthermore, in multipotent hematopoietic progenitors, EPC1 operates downstream of JNK to initiate cellular differentiation [55].

EPC1 and germ cell differentiation. EPC1 also promotes histone hyperacetylation during spermatid differentiation. This process is fundamental because as mentioned, it allows for the replacement of histones with transition proteins and protamines, which ultimately leads to genome compaction during spermiogenesis. Genetic ablation of either Epc1 or TIP60 disrupts this hyperacetylation process, impairing histone replacement and causing aberrant spermatid development. This acetylation creates a nuclear gradient, with faster acetylation near the apical pole and slower in the caudal regions that are needed for spermatid nucleus maturation [24].

EPC1 and HSCs. EPC1 regulates hematopoietic stem and progenitor cell (HSPC) development by linking epigenetic modulation to metabolic control. Loss of EPC1 or its paralog EPC2 impairs HSPC proliferation and reduces histone H3 acetylation, impacting hematopoiesis. Among downstream targets, DLST (dihydrolipoamide S-succinyltransferase), an essential enzyme in the tricarboxylic acid (TCA) cycle, shows expression patterns closely tied to histone H3 acetylation. This regulation appears to be mediated through EPC1’s collaboration with chromatin modifiers such as KATs and BRPF1. Since the TCA cycle is known to support both the emergence and differentiation of HSPCs, EPC1 likely serves as an integrator of metabolic activity and chromatin state. Furthermore, EPC1 may enhance DLST transcription by recruiting transcription factors like SRF or FOXR2 to its promoter, suggesting a context-specific mechanism that fine-tunes gene expression to sustain blood stem cell formation and expansion [56].

EPC1 and myoblast differentiation. EPC1 helps initiate myoblast differentiation by promoting skeletal muscle lineage commitment. Identified as a novel binding partner of the transcriptional co-regulator Hop, EPC1 is transiently upregulated during the early stages of H9c2 myoblast differentiation, preceding the induction of myogenic transcription factors such as MYOG (myogenin), MYOD1, MYF5, and MYF6. Its expression correlates with morphological changes like cell elongation and myotube formation, which are significantly impaired when EPC1 is silenced and enhanced when it is overexpressed. The functional interaction between EPC1 and HOP appears essential for optimal activation of muscle-specific genes, likely through modulation of chromatin structure or transcription factor accessibility [57]. Moreover, EPC1 interacts with SRF, a regulator of muscle differentiation and cell survival, to activate SRF/SRE-dependent genes by recruiting p300, which in turn induces skeletal muscle differentiation [58].

### 2.6. RUVBL1 and Differentiation

RUVBL1, also known as Pontin is an evolutionarily conserved chromatin remodeling protein with ATPase and helicase functions that is involved in several complexes including INO80, SWR1, and TIP60. It forms hexamers and affects transcription, DNA repair and embryonic development, partly through its interactions with transcription factors like c-Myc and p53 [59].

RUVBL1 and ESCs. Pontin is involved in maintaining ESC identity by acting as a transcriptional coactivator for OCT4, a core pluripotency factor. Loss of Pontin severely disrupts ESC self-renewal and compromises the expression of genes needed for maintaining pluripotency. Genome-wide analyses reveal that Pontin and Oct4 share many targets, including protein-coding genes and a distinct group of long intergenic non-coding RNAs (lincRNAs) that help repress differentiation. Pontin binds to these genomic regions in an Oct4-dependent manner and is needed for recruiting the histone acetyltransferase p300 to promote transcription. Although Oct4 binding remains intact without Pontin, the lack of p300 recruitment impairs transcriptional activation and destabilizes the ESC state. Through coordinated control of both coding and non-coding genes, Pontin regulates chromatin-based networks that preserve ESC pluripotency [60].

Other studies in mice have shown that loss of Pontin results in failure of embryos to progress beyond the blastocyst stage, due to impaired proliferation of the pluripotent inner cell mass. This suggests that Pontin helps sustain ESC viability and pluripotency, likely through its role in regulating transcriptional programs and chromatin structure necessary for stem cell function [61].

Pontin acts in retinoic acid (RA)-induced differentiation of embryonic stem cells by acting as a transcriptional co-activator. Upon RA stimulation, pontin is recruited to RA response elements (RAREs) in coordination with RARα, particularly at the HOX gene clusters, which are key regulators of embryonic patterning and cell fate. This recruitment is associated with increased transcriptional activity, as evidenced by enhanced H3K36me3 marks at HOX loci. Depletion of pontin significantly reduces RA-driven HOX gene expression, indicating that pontin is needed for the full activation of RA-responsive developmental genes [59].

RUVBL1 and HSCs. In the mouse, pontin plays a role in the survival and function of HSCs. Studies using conditional deletion of the gene encoding pontin (Ruvbl1) in hematopoietic tissues revealed that its absence leads to bone marrow failure due to loss of HSCs by apoptosis. This role of pontin in HSCs parallels its function in early embryonic development, where it supports the proliferation of pluripotent cells. Moreover, pontin’s interaction with factors like c-Myc may contribute to its function in sustaining the hematopoietic system, highlighting its importance in stem cell maintenance and potential implications for diseases such as cancer [61].

RUVBL1 and epidermal differentiation. Pontin also supports the survival and differentiation of basal epidermal cells during embryogenesis. Conditional deletion of Ruvbl1 in the mouse epidermis leads to profound defects in skin architecture, including the near-complete loss of the keratin-14-positive basal layer and failure to form skin appendages such as hair follicles. These abnormalities are evident as early as embryonic day 12.5 and culminate in severe skin barrier dysfunction and perinatal lethality. While the precise molecular mechanisms remain unclear, the phenotype suggests disrupted cell proliferation and survival, potentially involving impaired regulation of the p53 signaling pathway and transcriptional dysregulation of epidermal differentiation programs [62].

### 2.7. RUVBL2 and Differentiation

Reptin, a member of the AAA+ ATPase family (ATPases associated with diverse cellular activities), contains conserved Walker A and B motifs that bind ATP, arginine fingers, sensor domains, and shares limited homology with the bacterial RuvB ATP-dependent DNA helicase. Frequently found in complex with pontin (RUVBL1), reptin (RUVBL2) can also function independently or even exert opposing effects on gene regulation compared to pontin. Reptin interacts with transcription factors, co-regulators, and SUMO-modifying enzymes, thereby contributing to transcriptional regulation, DNA damage repair, and ribonucleoprotein complex biogenesis [63]. Moreover, reptin regulates oncogenic signaling by interacting with key oncogenic proteins such as β-catenin and c-Myc, influencing cancer cell proliferation and survival [64].

RUVBL2 and ESCs. RUVBL2 helps maintain pluripotency and self-renewal in hESCs. Its expression is significantly higher in hESCs than in mesenchymal stem cells, indicating a strong link to the unique properties of embryonic cells. As part of chromatin remodeling complexes, RUVBL2 interacts with β-catenin, c-Myc, and pontin (RUVBL1), regulators of gene expression programs that preserve the undifferentiated state. Its elevated levels support active Wnt signaling and genomic stability, suggesting that RUVBL2 not only marks the embryonic state but also acts as a regulator of stem cell plasticity and lineage decisions, providing insights into potential strategies for controlling differentiation and improving regenerative therapies [65].

RUVBL2 and NSCs. RUVBL2 also directs embryonic stem cells toward a neuroectodermal fate. Overexpression of RUVBL2 in mouse embryonic stem cells promotes neuroectodermal differentiation by upregulating specific markers such as Pax6 and nestin, while not inducing mesodermal or endodermal markers. This effect is observed during both spontaneous and directed differentiation, indicating a strong tendency toward neural lineage specification. In vivo, RUVBL2 is predominantly expressed in the ectodermal regions of early mouse embryos, further supporting its involvement in neuroectoderm development [66]. Similarly, during neural differentiation of human embryonic stem cells, RUVBL2 expression increases significantly and is accompanied by enhanced interactions with nuclear partners such as pontin, β-catenin, and ATF-2. These interactions likely contribute to transcriptional regulation and the chromatin remodeling required for neural commitment, suggesting a conserved role for RUVBL2 across species in promoting early neural and neuroectodermal differentiation [67].

RUVBL2 and germ cell differentiation. RUVBL2 contributes to germ cell differentiation primarily through post-transcriptional regulation. In round spermatids, it is part of an RNA-binding proteome that controls mRNA stability and prevents premature translation of key transcripts. This ensures that specific mRNAs remain repressed or properly processed until later stages of spermatid maturation, allowing accurate timing of protein synthesis required for elongation and fertility. While these functions are essential for spermatogenesis, current evidence indicates that RUVBL2 acts through RNA regulatory pathways rather than classical epigenetic mechanisms [68].

RUVBL2 and adipogenesis. RUVBL2 functions in both the nucleus and cytoplasm to support adipocyte development and metabolic function. As a direct downstream target of PPARγ, RUVBL2 expression is upregulated during 3T3-L1 pre-adipocyte differentiation, which in turn promotes adiponectin polymerization and secretion; key processes for improving insulin sensitivity and lipid metabolism [69]. Beyond its nuclear functions, RUVBL2 accumulates in the cytosol after differentiation, where it interacts with AS160 to enhance its phosphorylation. This interaction facilitates insulin-stimulated GLUT4 translocation to the cell surface, boosting glucose uptake into adipocytes and supporting energy storage. Altogether, by regulating adiponectin dynamics and insulin-responsive glucose transport, RUVBL2 reinforces both adipocyte maturation and metabolic health, underscoring its importance in adipose tissue development and insulin sensitivity [70].

### 2.8. JAZF1 and Differentiation

Juxtaposed with another zinc finger gene 1 (JAZF1) is a member of the C2H2-type zinc finger protein family that is recognized for its unique zinc finger domains facilitating specific DNA binding and transcriptional regulation. Initially identified in endometrial stromal tumors, JAZF1 functions in various cellular functions such as cell proliferation, differentiation, apoptosis, and metabolic regulation [71,72]. Recent genome-wide ChIP-seq analyses reveal a strong association between JAZF1 and H2A.Z acetylation at more than 1000 regulatory sites, primarily within intronic regions. Beyond its established link to tumorigenesis, JAZF1 also regulates fundamental processes such as ribosome biogenesis. This regulatory activity appears largely cell-type independent, suggesting that JAZF1 acts as a broad, context-independent modulator of cellular function [73].

JAZF1 and adipogenesis. JAZF1 regulates adipose differentiation through its interactions with nuclear receptors and adipokines. During the differentiation of 3T3-L1 preadipocytes, JAZF1 expression rises markedly, paralleling adipocytokine visfatin levels, suggesting a functional link. JAZF1 enhances visfatin transcription indirectly by upregulating PPARα and PPARβ/δ, which in turn promote visfatin expression, as shown by experiments using receptor antagonists and siRNAs. While visfatin facilitates adipocyte differentiation and triglyceride accumulation, JAZF1 paradoxically reduces lipid accumulation in mature adipocytes, likely by suppressing TAK1 and PPARγ expression and by promoting pathways that favor fatty acid oxidation through PPARα and PPARβ/δ activation [74,75].

Studies using mouse embryonic fibroblasts and 3T3-L1 cells have shown that loss of JAZF1 severely disrupts the formation of mature fat cells, indicating its role in proper adipose development. In mice with partial JAZF1 deficiency, reduced fat mass and altered glucose regulation further confirm its importance [76].

JAZF1 and myoblast differentiation. JAZF1 plays a dual role in skeletal muscle development by encouraging myoblast proliferation and hindering their differentiation into mature muscle fibers. Mechanistically, JAZF1 represses expression of transcription factors such as myocyte enhancer factor 2C (MEF2C) and myogenic regulatory factor (MRF4), which are crucial for activating myogenic gene programs and maintaining muscle fiber integrity. Additionally, JAZF1 indirectly reduces AMPD1 expression through these factors, affecting downstream AMPK signaling and potentially linking muscle function to metabolic regulation. Overexpression of JAZF1 leads to sustained cell cycle progression and delays differentiation, suggesting a possible role in muscle-related tumorigenesis when aberrantly regulated. Conversely, reducing JAZF1 levels accelerates myogenic gene expression, affecting muscle cell fate [77].

JAZF1 and multiciliated cell differentiation. The airway mucociliary escalator that is essential for respiratory health, depends on a balance between secretory and multiciliated cells to clear inhaled debris. Disruptions in this system can lead to chronic infections and serious lung diseases. JAZF1 plays a significant role in promoting multiciliated cell differentiation, as demonstrated through overexpression and knock-down experiments in mouse airway models. Together with Fibronectin type 3 and ankyrin repeat domains 1 (Fank1), JAZF1 acts to regulate this process. JAZF1 and Fank1 likely function downstream of IL6 signaling and upstream of the multiciliated cell transcription factor Foxj1, emphasizing roles in coordinating multiciliogenesis [78].

JAZF1 and β-cell differentiation. Disruption of JAZF1 in iPSCs derived from knockout mice leads to reduced size and impaired differentiation of insulin-producing β-cells, resulting in decreased insulin production and insulin resistance [72]. Although JAZF1 deficiency does not markedly affect early iPSC maintenance, it alters differentiation and proliferation capacity, as evidenced by smaller teratomas and lower expression of proliferation markers. Overexpression studies in various cell types, including cancer and cardiac microvascular cells, suggest that JAZF1 can promote proliferation through the Akt signaling pathway. In iPSCs, JAZF1 deficiency also leads to lower insulin and C-peptide levels in differentiated β-cells, accompanied by changes in key endoderm markers such as GATA6 and α-fetoprotein, which are critical for proper β-cell development. These findings indicate that JAZF1 regulates endodermal gene expression during β-cell differentiation, thereby influencing pancreatic insulin secretion, glucose homeostasis, and overall β-cell function in mice [79].

JAZF1 and decidualization. Human endometrial stromal cells undergo decidualization, a process required for implantation and pregnancy maintenance. In low-grade endometrial stromal sarcoma, the JAZF1-SUZ12 fusion protein disrupts PRC2 composition, reduces H3K27me3, increases H4 acetylation, and dysregulates decidual gene expression, contributing to tumorigenesis [80]. More recently, studies of wild-type JAZF1 demonstrated that it safeguards hESC survival and decidualization by repressing G0S2 transcription through inhibition of the transcription factor Purβ. Loss of JAZF1 leads to excessive apoptosis and impaired decidualization, defects also observed in recurrent spontaneous abortion patients [81]. Together, these findings suggest that JAZF1 contributes to decidualization both in pathological contexts through fusion events and under physiological conditions by regulating transcriptional programs that control cell survival.

JAZF1 and Treg differentiation. JAZF1 and PPAR-γ were examined for their roles in Treg differentiation, inflammation, and insulin resistance using a transgenic mouse model [82]. Notably, JAZF1 transgenic mice exhibited markers indicative of improved insulin sensitivity, compared to wild-type mice. Furthermore, JAZF1 overexpression correlated with elevated levels of CD4+, CD25+, and FOXP3+ markers associated with Treg differentiation, while PPAR-γ inhibition led to reduced expression of these markers. Inflammatory markers (TNF-α, IL-1β, IL-6) were decreased in JAZF1 transgenic groups, with concomitant increases in anti-inflammatory cytokines (IL-10, TGF-β). Conversely, PPAR-γ inhibition reversed these trends. The collaborative action of JAZF1 and PPAR-γ was implicated in promoting Treg differentiation and regulating insulin resistance by modulating pro- and anti-inflammatory cytokine expression. These findings suggest a synergistic role for JAZF1 and PPAR-γ in mitigating insulin resistance, suggesting potential as therapeutic targets for metabolic diseases.

### 2.9. TRRAP and Differentiation

The Transformation/Transcription Domain-Associated Protein (TRRAP) is a 3859 amino acid protein with a molecular weight of 434 kD in the Ataxia-Telangiectasia Mutated (ATM) superfamily [83]. TRRAP is a phosphoinositide 3 kinase-related kinase (PIKK) and a pseudokinase as it lacks the typical catalytic residues found in related kinases [84]. TRRAP is typically found as a monomer but is also a component of larger macromolecular assemblies, including the Spt-Ada-Gcn5 acetyltransferase complex (known as SAGA in yeast and mammals) and the nucleosome acetyltransferase of H4 complex (NuA4 in yeast and TIP60 in mammals). In mammals, TRRAP interacts with the transcription factors E2F1, c-Myc, p53, LXR, FoxO3, and β-catenin, resulting in the activation of their target genes [85,86]. Mechanistically, in collaboration with additional complex components, TRRAP recruits HATs such KAT5 and transcription factors (TFs) to chromatin, leading to increased histone acetylation and the initiation of target gene transcription [83].

TRRAP and HSCs. Conditional TRRAP deletion in bone marrow stem/progenitor cells revealed that TRRAP removal led to a depletion of hematopoietic stem and progenitor cells, paradoxically without impacting the stem cell niche [87]. TRRAP-deficient hematopoietic stem/progenitor cells exhibited increased apoptosis independent of p53, indicating that the loss of these cells caused bone marrow failure, resulting in the severe phenotype and high mortality in TRRAP-conditional knockout (CKO) mice. This suggests that TRRAP may regulate genes within the hematopoietic stem cell niche that are essential for the survival of stem/progenitor cells. TRRAP overexpression also increased the stability of the NANOG protein by inhibiting its ubiquitination that is typically regulated by FBXW8, an E3 ubiquitin ligase. Since NANOG is essential for maintaining the cancer stem cell (CSC)-like traits of colon cancer spheroids [88], TRRAP may promote cancer spheroid development.

The Wnt pathway is vital for embryonic development and stem cell self-renewal, but excessive activation is associated with various human cancers. Central to this pathway is β-catenin. TRRAP interacts with Skp1/SCF, promoting the ubiquitination of β-catenin. Deletion of TRRAP reduces β-catenin ubiquitination, slows degradation, and causes β-catenin accumulation, ultimately leading to hyperactive Wnt signaling [89].

TRRAP and neuronal differentiation. The TRRAP-HAT-Sp1 axis also influences neural arborization and protects neural cells by regulating the Sp1 pathway, which in turn governs microtubule dynamics [90]. Deleting TRRAP has detrimental effects on the self-renewal and differentiation capacity of adult neural stem cells (aNSCs) in both mice and cell cultures. Deacetylating the K639 residue of Sp1 allowed Sp1 to bind to chromatin at target gene promoters, a process normally hindered by TRRAP. In the absence of TRRAP, K639 on Sp1 becomes acetylated by HATs independent of TRRAP, preventing Sp1 from binding to chromatin and repressing gene expression related to adult neurogenesis. Additionally, TRRAP deletion destabilizes Sp1, reducing its transcription-activating capability. This destabilization may be attributed to the deacetylation of other residues influenced by TRRAP-HAT that have yet to be identified, or because TRRAP itself is necessary for maintaining Sp1’s stability as a scaffold [91].

TRRAP and ESCs. Depleting TRRAP in ESCs led to unforeseen differentiation, likely attributed to TRRAP’s critical role in HAT-mediated chromatin remodeling and its regulation of essential stemness marker genes such as Nanog, Oct4, and Sox2. Maintaining high TRRAP levels impedes ESC differentiation, underscoring the importance of reducing TRRAP expression for transcriptional reprogramming and ESC differentiation [83].

TRRAP and multiciliated cell differentiation. TRRAP operates between the Notch2-mediated determination of basal progenitor cell fate and the subsequent regulation of Multicilin to control the differentiation of multiciliated cells (MCCs). It binds to promoters and controls the expression of a set of genes involved in MCC differentiation and function, including several genes associated with human ciliopathies [92]. Through a kinome-wide RNA interference approach, TRRAP was also identified as a regulator of brain tumor-initiating cell (BTIC) differentiation. Knocking down the expression of the adaptor protein TRRAP significantly promoted BTIC differentiation in culture, sensitized the cells to apoptotic signals, and negatively affected cell cycle progression [93]. Down regulation of TRRAP-dependent hTERT expression by Myc/Max was also found to contribute to the differentiation of HL60 cells after exposure to DMSO [94].

### 2.10. DMAP1 and Differentiation

Dnmt1-associated protein 1 (DMAP1), one of the components of both the NuA4 and Swr1/SRCAP complexes, was originally identified as a molecule interacting with DNMT1 that co-localized with PCNA and DNMT1 at DNA replication foci during S phase in human cells. The DMAP1–DNMT1 complex was also reported to interact with the p33ING1–Sin3–HDAC complex and localize in pericentric heterochromatin to maintain heterochromatin structure and histone modifications in late S phase, affecting cell differentiation capabilities [95,96].

DMAP1 and HSCs. The Mat1-mediated transcriptional repressor (MMTR)/DMAP1 is a transcription corepressor involved in chromatin remodeling, cell cycle regulation, DNA double-strand break repair, and tumor suppression. DMAP1 functions in the repair of damaged DNA in mammals that is linked to stem cell aging, causing HSC depletion over time. DMAP1-knockdown induced by DMAP1-specific shRNA severely compromised the proliferative capacity of HSCs in vitro and long-term repopulating capacity of HSCs in recipient mice [97].

DMAP1 and ESCs. MMTR/DMAP1, along with other TIP60-EP400 complex proteins, binds the promoters of differentiation commitment genes in mouse ESCs and controls gene expression during differentiation [98]. A Dmap1-null allele was generated to study the role of DMAP1 in development. The results suggest that Dmap1−/− mice died during preimplantation and the DMAP1 somatic form of DNA methyltransferase 1 (DNMT1s) and the DMAP1 oocyte form (DNMT1o) were needed for normal development [99]. An RNA interference (RNAi) screen performed in mouse ESCs showed that reduction in the expression of individual components of the TIP60-EP400 complex, including DMAP1, caused a loss of characteristic ES cell morphology and activation of genes associated with cell differentiation [100].

DMAP1 and myoblast differentiation. Utilizing CRISPR-KO screening, Yu et al. uncovered a role for DMAP1 in mediating both progenitor induction and differentiation of chemically induced smooth muscle progenitor cells via regulation of the methylation of the promoters of Nkx2–5 and Cdh1, respectively [101].

### 2.11. YEATS Family and Differentiation

The YEATS domain is a conserved module that recognizes histone acylation. It was first described in a group of five proteins (Yaf9, ENL, AF9, Taf14, and Sas5) and is now found in more than 100 proteins across more than 50 organisms. In humans, the best-studied YEATS proteins are AF9 (MLLT3), ENL, and YEATS4/YAF9/Gas41 [102]. The AF9 protein interacts with DOT1L, MLL, and MPc3, and affects cell differentiation. Different studies suggest that mutations in the human AF9 gene are linked to neurodevelopmental disorders, such as intellectual disability, epilepsy, and ataxia [103,104].

### 2.12. YEATS Family and Neuronal Differentiation

Although not part of the TIP60/NuA4 complex, YEATS-domain proteins such as AF9 (MLLT3) highlight the broader role of this family in lineage commitment. AF9 preferentially binds acetylated H3K9, with weaker affinity for H3K27ac and H3K18ac, and interacts with DOT1L and MLL complexes to regulate transcriptional programs [105]. In neural differentiation, AF9 cooperates with TET2 at neurodevelopmental loci, where it promotes conversion of 5mC to 5hmC and activation of neuronal genes [106]. Loss of Af9 disrupts Hox regulation and corticogenesis [107,108], and human AF9 mutations are associated with intellectual disability, epilepsy, and ataxia [103,104]. Together, these findings highlight the importance of YEATS readers in neural fate decisions, even though their role is independent of NuA4.

YEATS family and ESCs. By contrast, YEATS4/YAF9/Gas41 is the only YEATS protein directly incorporated into the TIP60/NuA4 complex. Its yeast homolog, Yaf9, occupies the same position in the NuA4 complex of Saccharomyces cerevisiae, underscoring its conserved function across species. Both YEATS4 and Yaf9 share a domain organization with an N-terminal YEATS domain and C-terminal A/B boxes. Functional studies in mESCs show that YEATS4 preserves pluripotency by repressing developmental genes: knockdown induces flattened morphology, reduced alkaline phosphatase activity, and enrichment of embryoid body differentiation signatures [109,110]. These results suggest that YEATS4/YAF9/Gas41 acts as a conserved scaffold and chromatin reader within TIP60/NuA4, anchoring the complex to histone acylation sites and preventing premature differentiation.

### 2.13. BAF53A/ACTL6A and Differentiation

The BRG1/BRM-associated factors (BAF) complex, alternatively recognized as the mammalian SWI/SNF ATP-dependent chromatin-remodeling complex, plays a role in influencing the differentiation of both embryonic and adult stem cells. ACTL6A (also known as BAF53A or ARP4) is a shared subunit present in multiple complexes, such as esBAF, npBAF, INO80, and TIP60/NuA4 complex. Its expression is observed in various stem/progenitor cells, including neural progenitor cells, hematopoietic stem cells, epidermal progenitor cells, and ESCs [111,112].

BAF53A/ACTL6A and ESCs. BAF53A/ACTL6A can sustain a progenitor state in epidermal cells by inhibiting klf4, an activator of differentiation. Conditional knockout (cKO) of the BAF53A/ACTL6A gene in mouse epidermal cells resulted in cell cycle exit, terminal differentiation, and hypoplasia, whereas ectopic expression of BAF53A/ACTL6A repressed Klf4 expression and enhanced the epidermal progenitor state [113]. BAF53A/ACTL6A protects mESCs from differentiating into primitive endoderm (PrE). Specifically, BAF53A/ACTL6A can interact with Nanog and Sox2, facilitating Nanog’s binding to pluripotency-related genes like Oct4 and Sox2. Moreover, BAF53A/ACTL6A is able to bind to the promoters of PrE regulators like Sall4 and Fgf4, suppressing their expression and impeding the differentiation of mESCs into primitive endoderm [114]. In hypoxic conditions, HIF-1α, a subunit of hypoxia-inducible factor, preserves the pluripotency of pluripotent stem cells (PSCs). Cui and colleagues [115] discovered that disrupting HIF-1α expression led to a decline in self-renewal and pluripotency in human induced pluripotent stem cells (hiPSCs). The knockdown of HIF-1α hindered the expression of BAF53A/ACTL6A and the acetylation of histone H3K9 (H3K9ac). Knocking down BAF53A/ACTL6A resulted in reduced levels of H3K9ac and compromised pluripotency in hiPSCs, impacting their differentiation into endoderm. BAF53A/ACTL6A exhibited increased expression in fully pluripotent stem cell lines. ATP stimulation enhanced both BAF53a/ACTL6A expression and histone acetylation. Knocking down BAF53a/ACTL6A resulted in decreased pluripotency in ESCs, and the addition of ATP did not restore this reduction. Additionally, suppressing ATP production through rotenone treatment diminished the pluripotency of ESCs. Together, these findings indicate that the level of mitochondrially generated ATP influences stem cell pluripotency through BAF53A/ACTL6A-mediated histone acetylation [116].

BAF53A/ACTL6A and neuronal differentiation. Intellectual disability is linked to heterozygous variations in BAF53A/ACTL6A [51]. Baf45a and BAF53A/ACTL6A function as subunits within the neural stem/progenitor BAF (npBAF) complex in neural stem/progenitor cells. As neural progenitors exit the cell cycle, these subunits are further replaced by the analogous BAF45B, BAF45C, and BAF53B proteins. The presence of BAF45A/53A subunits is essential for both the initiation and continuation of neural progenitor proliferation. Disrupting the switch between these subunits hampers the process of neuronal differentiation [117].

Yoo and colleagues demonstrated that the transition is facilitated through the suppression of BAF53A/ACTL6A by miR-9* and miR-124. They identified that the repression of BAF53A/ACTL6A is governed by specific sequences in the untranslated region, corresponding to the recognition sites for miR-9* and miR-124, which are selectively active in post-mitotic neurons. The mutation of these sites resulted in the sustained expression of BAF53A/ACTL6A and impaired activity-dependent dendritic outgrowth in neurons. Moreover, the overexpression of miR-9* and miR-124 in neural progenitors led to reduced proliferation [118].

Park and coworkers recognized the significance of BAF53A/ACTL6A in linking the recognition of axonal caliber with the transcriptional program of myelination. Exposure to large caliber axons or nanofibers enhances the nuclear levels of BAF53A/ACTL6A in Schwann cells (SC), leading to the removal of repressive histone marks and thereby promoting myelination. In the absence of BAF53A/ACTL6A, Schwann cells are unable to synchronize caliber recognition and the production of myelin [119].

BAF53A/ACTL6A and HSCs. The conditional knockout (cKO) of BAF53A/ACTL6A in HSCs led to mice experiencing bone marrow failure, aplastic anemia, and death. Analysis of cell counts revealed a reduction in mature hematopoietic cells, including macrophages, granulocytes, erythrocytes, B cells, and T cells, as well as in HSCs/progenitor cell fractions such as common myeloid progenitors, megakaryo-erythrocyte progenitors, granulocyte-monocyte progenitors and so on in the bone marrow of BAF53A/ACTL6A cKO mice. These findings indicate the involvement of BAF53A/ACTL6A in the proliferation and survival of hematopoietic cells [120].

The long non-coding RNA uc.291, containing a highly conserved element, was demonstrated to engage in a physical interaction with BAF53A/ACTL6A, influencing chromatin remodeling for the facilitation of cellular differentiation. When uc.291 was depleted, BAF53A/ACTL6A exhibited binding to promoters of differentiation genes, impeding the BAF complex from targeting them and thereby hindering the activation of terminal differentiation genes. Conversely, in the presence of uc.291, the inhibitory effect of BAF53A/ACTL6A was alleviated, enabling chromatin modifications that foster the expression of differentiation genes [121].

### 2.14. EP400 and Differentiation

EP400/mammalian Domino, which was identified as an interaction partner for adenovirus E1A and the myeloid-specific transcription factor MZF-2A, is an SWR1-class chromatin-remodeling ATPase that is homologous to the yeast Swr1p and Drosophila Domino proteins. The EP400 protein, like its homologs in other eukaryotes, catalyzes ATP-dependent incorporation of histone H2A variant H2A.Z into chromatin via exchange of H2A-H2B dimers within nucleosomes for free H2A.Z-H2B dimers. It can also incorporate histone H3 variant H3.3 into chromatin. H2A.Z and H3.3 are often enriched near gene regulatory regions, consistent with a role for EP400 (like TIP60) as a co-activator of transcription. However, EP400 also appears to repress transcription in some contexts as well as promote DNA repair in concert with TIP60 [27,122].

EP400 and neuronal differentiation. Elsesser and coworkers targeted the deletion of EP400 during distinct phases of mouse oligodendrocyte development. Despite the normal development of EP400-deficient oligodendrocyte precursors, significant impairment was observed in terminal differentiation and myelination processes. This impairment is attributed to the mechanistic involvement of EP400, which interacts with the transcription factor Sox10, binds to regulatory regions of the Myrf gene, and is essential for inducing this central transcriptional regulator within the myelination program [123]. Moreover, the deletion of EP400 specifically in Schwann cells (SCs) in mice led to defects in the late stages of SC development and peripheral myelination, stemming from the aberrant expression of developmental regulators [124].

EP400 and HSCs. The targeted mutation of EP400/mDomino in mice resulted in early embryonic lethality accompanied by significant deficiencies in yolk sac hematopoiesis. In embryos with the mDomino mutation, there was a notable decrease in the expression of embryonic globin genes and a globin chaperone gene. Conversely, there was a substantial increase in the expression of a specific set of Hox genes. These findings indicate a role for EP400/mDomino in the epigenetic regulation of genes under developmental control [125]. Fujii and colleagues explored the involvement of the EP400/mDomino gene in adult bone marrow hematopoiesis and embryonic fibroblast cell-cycle progression by generating a conditional knock-out mouse model. This led to a sudden decline in nucleated cells within the bone marrow, affecting committed myeloid and erythroid cells, as well as hematopoietic progenitor and stem cells. Additionally, the absence of EP400/mDomino in MEFs resulted in pronounced growth inhibition. Analysis of DNA microarray uncovered that the deletion of EP400/mDomino from MEFs led to impaired expression of numerous cell-cycle-regulatory genes, including G2/M-specific genes regulated by the transcription factors FoxM1 and c-Myc [122].

EP400 and adipogenesis. Following hormonal stimulation, the expression of Brd8, EP400, TIP60, H2A.Z, and PPARγ increases in 3T3-L1 preadipocytes. Subsequently, the PPARγ/RXRα heterodimer forms a complex with Brd8 and EP400, binding to PPAR-response elements (PPREs). In this process, EP400 incorporates H2A.Z into chromatin, leading to transcriptional activation by recruiting RNAPII. Additionally, TIP60 is recruited to the complex by Brd8, promoting histone acetylation and thereby further enhancing transcription [126].

## 3. Whole-Complex Perspective of TIP60/NuA4 in Differentiation

The TIP60/NuA4 complex is a unified chromatin-remodeling entity that integrates enzymatic, structural, and reader activities to regulate gene expression during differentiation. Individual subunits perform specific roles such as histone acetylation by TIP60/KAT5 and histone-variant exchange by EP400, but the complex orchestrates these functions to create a chromatin environment favorable for lineage specification. EP400 and scaffolding subunits such as EPC1 act as organizing hubs, assembling catalytic and reader modules and positioning TIP60/KAT5 alongside histone mark-binding proteins including ING3, MBTD1, and MRG15. By coupling chromatin-state recognition with enzymatic activities such as histone acetylation and H2A.Z incorporation, TIP60/NuA4 ensures precise transcriptional regulation [7].

This integrated activity translates into distinct lineage-specific outcomes. TIP60-mediated H2A.Z acetylation promotes H3K4me3 deposition and bivalent gene activation, enabling neuronal fate commitment [15]. In muscle, TIP60 cooperates with MyoD to drive myogenic transcriptional programs [28], while in adipogenesis, TIP60 enhances PPARγ activity to promote adipocyte differentiation [31]. In hematopoietic stem cells, TIP60 and TRRAP interact with Myc to sustain proliferation and survival [22,87]. In germ cells, TIP60/NuA4 is essential for gametogenesis, with reader subunits such as ING3 and MRG15 likely contributing to locus-specific targeting. Loss of scaffolding or catalytic components, such as EP400 or TIP60, destabilizes the complex and disrupts these processes, underscoring the importance of its integrity for proper lineage specification.

Overall, TIP60/NuA4 functions as a cohesive epigenetic hub that simultaneously reads histone marks, catalyzes acetylation, and exchanges histone variants to shape a chromatin landscape permissive for lineage-specific gene expression. This whole-complex perspective explains why different subunits recur across diverse developmental systems and why disruption of distinct components often produces overlapping differentiation defects. In essence, TIP60/NuA4 operates as an orchestrator of chromatin-state transitions, ensuring that progenitor cells activate the appropriate transcriptional programs at the right developmental stage [127].

### 3.1. Therapeutic Outlook

Beyond its fundamental role in chromatin regulation, TIP60/NuA4 is increasingly viewed through a translational lens. Dysregulation of TIP60 has been linked to neurodegeneration, cancer, cardiac injury, and immune imbalance. Context-dependent modulation, either enhancing or inhibiting TIP60 activity, may open therapeutic avenues, but dosing, timing, and genomic stability considerations are critical.

### 3.2. Neurodegeneration

Preclinical models show that loss of TIP60 reduces histone acetylation, represses neuroplasticity genes, and impairs cognition. Enhancing TIP60 activity via genetic overexpression or small-molecule activators restores synaptic function and neuronal gene programs and mitigates behavioral deficits, suggesting that this targeted alternative to broad HDAC inhibition may be more effective [128].

### 3.3. Oncology

TIP60 (KAT5) participates in DNA-damage signaling through ATM/p53 acetylation, checkpoint control, and context-specific transcription. Prototype TIP60 inhibitors such as NU9056, TH1834 and MG149 show anti-tumor effects or enhance DNA-damage sensitivity in models; conversely, in settings where TIP60 is downregulated, preserving or restoring TIP60 function may be beneficial. Biomarker-guided strategies examining TIP60 activity, ATM signaling and HR competence will likely be required for TIP60-directed therapies to be effective [129].

### 3.4. Cardiac Injury

In mice, initiating use of the TH1834 selective TIP60 inhibitor a few days after myocardial infarction improves contractile function, lowers apoptosis, and increases cardiomyocyte cell-cycle markers. These data support time-restricted TIP60 inhibition to promote repair while avoiding prolonged suppression of genome-maintenance pathways [130].

### 3.5. Immune Regulation

TIP60 also cooperates with p300 to acetylate FOXP3 and stabilize Treg identity and function. Treg-specific deletion of TIP60 depletes peripheral Tregs and causes severe autoimmunity. In contrast, p300-mediated autoacetylation of TIP60 at K327 promotes TIP60 stability and FOXP3 acetylation, highlighting TIP60 as a potential target to enhance immune tolerance [131].

### 3.6. Translational Outlook

Because TIP60/NuA4 controls lineage-specific transcriptional programs, enhancing TIP60 may improve derivation of neurons or myotubes from iPSCs, whereas transient inhibition can help preserve pluripotency (with caution due to DNA-repair roles). Subunit-focused approaches such as ligands targeting the ING3 PHD–H3K4me3 interface or modulators of TRRAP-dependent pathways offer testable routes for tissue engineering and targeted therapy.

## 4. Gaps and Controversies

TIP60/NuA4 regulates multiple developmental programs. However, significant gaps remain in understanding how its subunits collaborate to resolve bivalency during lineage transitions.

Bivalent chromatin—marked by both H3K4me3 and H3K27me3—was once thought to prime developmental genes for rapid activation. Instead, recent studies suggest its main role is protective: H3K4me3 repels de novo DNA methyltransferases, keeping promoters accessible and plastic. Loss of this bivalency in cancer and aging predisposes CpG-island promoters to aberrant hypermethylation and irreversible silencing [132].

Although TIP60/NuA4 contributes to diverse lineage transitions, including myogenic and hematopoietic differentiation, the neuronal system currently provides the most detailed mechanistic evidence. In neuronal differentiation, TIP60 (KAT5) plays a decisive role in resolving bivalency toward activation. Its chromodomain first guides the complex to bivalent promoters, where TIP60 acetylates H2A.Z. This acetylation enables de novo H3K4me3 deposition at lineage-restricted genes such as Miat, driving their transcriptional activation. Without TIP60 or H2A.Z acetylation, these promoters fail to acquire H3K4me3 and remain silent, stalling neuronal fate transitions [15]. Structure–function studies confirm that TIP60’s acetyltransferase activity and chromodomain recognition are indispensable for neurogenesis, whereas other domains (NR-box, zinc finger) appear less critical [15]. While neurons provide the clearest mechanistic model so far, whether similar principles apply to other lineages such as myogenic or hematopoietic systems remains an open question.

A key unresolved question is what happens once H3K4me3 is deposited. Does the complex “handoff” chromatin recognition from the KAT5 chromodomain to the ING3 PHD finger, which specifically binds H3K4me3? If so, such a switch could stabilize the complex at newly activated promoters or alter its enzymatic activity toward different histone substrates. While this remains hypothetical, a staged reader engagement could provide a dynamic mechanism for guiding ESCs through successive steps of neuronal differentiation—from initial targeting of silent bivalent loci to reinforcement of active neuronal promoters.

During DNA repair, TIP60 also acts as a potential “bivalency resolver.” It disrupts 53BP1’s dual recognition of H4K20me and H2AK15ub through MBTD1-mediated binding and H2AK15 acetylation, thereby promoting homologous recombination. Its own post-translational modifications—including phosphorylation, autoacetylation, SUMOylation, and ubiquitination—fine-tune this activity [133,134]. Drawing parallels between DNA repair and differentiation could reveal common rules by which TIP60 dismantles incompatible chromatin states.

Another unresolved issue is whether TIP60/NuA4 subunits always act as part of the holo-complex or can exert independent functions. RUVBL2, for instance, contributes to RNA splicing outside the TIP60/NuA4 complex, and ING proteins have been implicated in other chromatin contexts. This raises the possibility that some “reader” functions attributed to TIP60/NuA4 may, under certain conditions, occur independently of the complex. Clarifying this distinction is critical for determining whether TIP60’s roles in differentiation are strictly complex-dependent or partly modular.

Another important gap is how TIP60/NuA4 coordinates with other chromatin regulators. Complexes such as p300/CBP and SWI/SNF (BAF) are also engaged at lineage-specific enhancers, and it remains unclear whether TIP60 collaborates with or competes against these regulators during fate transitions. Addressing this will help place NuA4 within the broader network of chromatin-remodeling machinery.

Methodologically, most studies to date have relied on static knockout or knockdown approaches. New tools such as inducible degrons, domain-specific mutants, and time-resolved chromatin profiling (CUT and Tag, CUT and RUN) will be essential to dissect stage-specific roles of TIP60 and its subunits across the ESC–NPC–neuron axis.

Finally, these mechanistic uncertainties have therapeutic implications. Because TIP60 activity integrates reader switching, subunit collaboration, and PTM regulation, targeted interventions may eventually allow stage-specific control of differentiation while avoiding the risks of global TIP60 inhibition. Dysregulation of TIP60 has also been linked to impaired differentiation in cancers and to synaptic defects in neurodegenerative disease, underscoring its relevance to both development and pathology. Such insights could guide future strategies for regenerative medicine and disease modeling.

## 5. Conclusions

TIP60/NuA4 is a central epigenetic hub that integrates reader-guided chromatin targeting, KAT5-mediated histone acetylation, and EP400-driven histone-variant exchange to regulate lineage-specific differentiation. Across neuronal, hematopoietic, germline, and mesenchymal systems, perturbing reader/ATPase/HAT modules yields convergent differentiation defects, underscoring functional interdependence. Nonetheless, key uncertainties remain: do lineage outcomes require the intact complex, or can separable submodules suffice? Evidence that ING3 (osteogenesis) and MBTD1 (HSC fate) act with TIP60 is compelling but not yet definitive, and RUVBL2’s role in spermatids appears post-transcriptional rather than epigenetic. Dissecting HAT-dependent vs. HAT-independent functions of TIP60/KAT5, defining subunit requirements for locus specificity (e.g., ING3, MRG15, MBTD1), and mapping potential cell type-specific “flavors” of the complex should be priorities. Ultimately, a deeper mechanistic map will not only refine our understanding of chromatin-state transitions but also enable biomarker-guided, subunit-selective interventions. Such strategies could support regenerative medicine and yield targeted therapies for diseases rooted in defective differentiation, including leukemia, infertility, and neurodegeneration.

## Figures and Tables

**Table 1:** Human TIP60/NuA4 subunits, aliases, and functional categories. Subunits are organized by primary roles: catalytic, ATPase/remodeler, scaffold, reader, histone chaperone, or structural.

Subunit	Full Name	Aliases	General Function/Category
KAT5	Lysine Acetyltransferase 5	TIP60	Catalytic subunit; histone acetyltransferase (HAT)
EP400	E1A Binding Protein p400	p400	ATP-dependent remodeler and central scaffold; histone variant exchange
EPC1	Enhancer of Polycomb Homolog 1	Epl1 (yeast homolog)	Scaffold; organizes/links modules and anchors HAT components
RUVBL1	RuvB-Like AAA + ATPase 1	Pontin, TIP49	AAA+ ATPase; structural/regulatory core with RUVBL2
RUVBL2	RuvB-Like AAA + ATPase 2	Reptin, TIP48	AAA+ ATPase; structural/regulatory core with RUVBL1
ING3	Inhibitor of Growth Family Member 3	—	PHD-finger, reader-type subunit; HAT module component
MRG15	MORF4-Related Gene 15	MORF4L1	Chromodomain protein; reader-type subunit
MBTD1	Malignant Brain Tumor Domain-Containing 1	—	MBT-repeat reader; binds H4K20me
YEATS4	YEATS Domain-Containing 4	GAS41, YAF9 (yeast homolog)	YEATS-domain protein; reader-type subunit
TRRAP	Transformation/Transcription Domain-Associated Protein	—	Large scaffold; activator-binding hub (associates with EP400 SANT)
BRD8	Bromodomain-Containing Protein 8	—	Bromodomain subunit; links auxiliary components to EP400 N-terminus
DMAP1	DNMT1-Associated Protein 1	—	Auxiliary scaffold; stabilizes EP400 HSA/actin-related interfaces
ACTL6A	Actin-Like Protein 6A	BAF53A; ARP4 (yeast)	Actin-related subunit(s); two copies bound along EP400 HSA
VPS72	Vacuolar Protein Sorting-Associated Protein 72	YL1	Histone chaperone; contacts RUVBL1/2 and EP400
MEAF6	MYST/Esa1-Associated Factor 6	EAF6 (yeast homolog)	Small HAT subunit; stabilizer
MRGBP	MRG-Binding Protein	C20orf20	Accessory protein; MRG15 partner
ACTB	β-Actin	Beta-actin	Actin subunit; forms heterodimer with ACTL6A

## Data Availability

No new data were created or analyzed in this study. Data sharing is not applicable to this article.
